# Galectin-1 induces invasion and the epithelial-mesenchymal transition in human gastric cancer cells via non-canonical activation of the hedgehog signaling pathway

**DOI:** 10.18632/oncotarget.13201

**Published:** 2016-11-08

**Authors:** Yang Chong, Dong Tang, Jun Gao, Xuetong Jiang, Chuanqi Xu, Qingquan Xiong, Yuqin Huang, Jie Wang, Huaicheng Zhou, Youquan Shi, Daorong Wang

**Affiliations:** ^1^ Department of Gastrointestinal Surgery, Clinical Medical College of Yangzhou University, Subei People's Hospital of Jiangsu Province, Yangzhou 225001, China

**Keywords:** galectin-1, epithelial-mesenchymal transition, hedgehog signaling, Gli-1, gastric cancer

## Abstract

Galectin-1 (Gal-1) has been reported to be an independent prognostic indicator of poor survival in gastric cancer and overexpression of Gal-1 enhances the invasiveness of gastric cancer cells. However, the downstream mechanisms by which Gal-1 promotes invasion remains unclear. Moreover, the function of Gal-1 in the epithelial-mesenchymal transition (EMT) in gastric cancer has not yet been elucidated. In this study, we observed Gal-1 expression was upregulated and positively associated with metastasis and EMT markers in 162 human gastric cancer tissue specimens. *In vitro* studies showed Gal-1 induced invasion, the EMT phenotype and activated the non-canonical hedgehog (Hh) pathway in gastric cancer cell lines. Furthermore, our data revealed that Gal-1 modulated the non-canonical Hh pathway by increasing the transcription of glioma-associated oncogene-1 (*Gli-1*) via a Smoothened (SMO)-independent manner, and that upregulation of Gal-1 was strongly associated with gastric cancer metastasis. We conclude that Gal-1 promotes invasion and the EMT in gastric cancer cells via activation of the non-canonical Hh pathway, suggesting Gal-1 could represent a promising therapeutic target for the prevention and treatment of gastric cancer metastasis.

## INTRODUCTION

Gastric cancer is the fifth most common malignant tumor type and third-leading cause of cancer-related deaths worldwide [1]. Approximately half of all global deaths due to gastric cancer occur in East Asia, predominantly in China [1]. More than 90 percent of cancer-related deaths among patients with solid tumors are not the result of the primary tumor, but due to the metastasis and invasion of secondary tumors in different organs [2]. Metastatic progression, the spread of primary tumors to distant organs, is a complex, multistep physiological process. A large number of studies have shown the epithelial-mesenchymal transition (EMT) plays a critical important role in tumor cell invasion and metastasis, and leads to upregulation of mesenchymal genes such as Vimentin and downregulation of epithelial-associated markers such as E-cadherin [3]. The EMT occurs during tumor progression and confers carcinoma cells with a more aggressive phenotype [4]. As a result of the EMT, tumor cells acquire metastatic and invasive properties, exhibit characteristics that resemble embryonic mesenchymal cells, and have enhanced ability to penetrate the surrounding stroma to initiate the formation of new neoplastic foci [4, 5].

Galectin-1 (Gal-1), encoded by the *LGALS1* gene, is a member of the carbohydrate-binding proteins family, which are characterized by their affinity for β-galactoside-containing glycans [6]. Gal-1 can participate in sugar-independent intracellular interactions with other proteins [7]. In the extracellular environment, Gal-1 can be activated by autocrine sugar-dependent and paracrine interactions with β-galactoside-containing glycoconjugates [8, 9]. It has been reported that increased Gal-1 expression is associated with tumor malignancy in a variety of human cancers [10–13], including gastric cancer [14], with positive associations demonstrated between high expression of Gal-1 and enhanced gastric cancer cell migration and invasion in vitro [15]. In addition, our previous studies showed Gal-1 was associated with poorer patient prognosis and could promote angiogenesis in gastric cancer [16].

It has been reported that Gal-1 promotes pancreatic carcinogenesis via activation of Hedgehog (Hh) signaling [17]. Hh signaling includes both the canonical and non-canonical signaling pathways [18]. Normally, the zinc finger transcription factors glioma-associated oncogene -1 (Gli-1) are activated by ligand binding of Patched (Ptch), a 12-pass transmembrane receptor of Sonic Hedgehog (SHH), leading to activation a transmembrane spanning protein called Smoothened (SMO); this is the canonical Hh signaling pathway [18]. However, in some situations, the Gli transcription factors can be activated by other molecules/signaling independently of the ligand SHH; this is termed non-canonical Hh signaling [18]. Non-canonical Hh signaling has been widely investigated in the context of malignant disease [18]. There is strong evidence that the Hh pathway is involved in the EMT in a range of malignant tumors, including gastric cancer [19, 20].

In this study, we investigated whether endogenous Gal-1 regulates the EMT by activating the Hh pathway in gastric cancer. We compared the expression of Gal-1 in cancer tissues and non-cancerous tissues of patients with gastric cancer and investigated the associations between Gal-1 expression and the clinicopathological features of patients with gastric cancer. Based on these clinical data, we performed in vitro experiments to assess the effects of upregulating or downregulating Gal-1 on the invasion and EMT in gastric cancer cell lines. This study suggests Gal-1 increases gastric cancer cell invasion and promotes the EMT by the activating the non-canonical Hh signaling pathway.

## RESULTS

### Upregulation of Gal-1 is clinically associated with the EMT and metastasis in human gastric cancer

**Figure 1:**
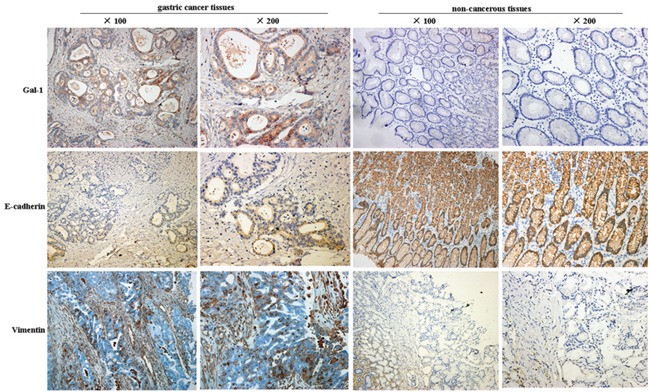
Representative images of immunohistochemical staining for Gal-1, E-cadherin and vimentin in human gastric cancer tissues and non-cancerous tissues

In order to elucidate the role of Gal-1 in gastric cancer, we first performed immunohistochemistry analyses of 162 paired gastric cancer tissues and non-cancerous tissues from patients with gastric cancer. Compared with the matched non-cancerous tissues, the gastric cancer tissues exhibited significantly higher expression of Gal-1 (Figure 1). Moderate Gal-1 staining was detected in the stroma of normal mucosa, while the Gal-1 staining intensity was significantly higher in the stroma and epithelium of the gastric cancer tissues. We then determined the associations between Gal-1 and the expression of E-cadherin and vimentin. As shown in Table 1, in most cases, the expression of Gal-1 and vimentin were significantly higher in the gastric cancer tissues than the matched non-cancerous tissues (*P* < 0.05). In contrast, the expression of E-cadherin was significantly lower in the gastric cancer tissues than the matched non-cancerous tissues (*P* < 0.05).

**Table 1:** Univariate analysis of galectin-1, E-cadherin and vimentin protein expression in 162 matched human gastric adenocarcinoma tissue samples

Protein	Gastric cancer tissues	Non-cancerous tissues	*P*-value
**Gal-1**			
**+**	96/162(59.26%)	48/162(29.63%)	0.009
**-**	66/162(40.74%)	114/162(70.37%)	
**E-cadherin**			
**+**	63/162(38.89%)	118/162(72.84%)	0.000
**-**	99/162(61.11%)	44/162(27.16%)	
**Vimentin**			
**+**	116/162(71.60%)	60/162(37.04%)	0.004
**-**	46/162(28.40%)	102/162(62.96%)	

The associations between the clinicopathological features of the patients with gastric cancer and the Gal-1 immunohistochemical staining score are summarized in Table 2. E-cadherin and vimentin were strongly associated with the depth of tumor invasion, lymph node metastasis and advanced TNM stage. As shown in Table 3, Gal-1 expression was negatively associated with E-cadherin expression but positively correlated with vimentin expression in gastric cancer. These results collectively suggest that Gal-1 may be closely associated with metastasis and the EMT in gastric cancer.

**Table 2:** Associations between Gal-1, E-cadherin and vimentin immunostaining and the clinicopathological features of 162 patients with gastric cancer cases assessed using the chi-square test

Parameters	n		Gal-1			E-cadherin			Vimentin	
		+	−	P value	+	−	P value	+	−	*P*-value
**Age (years)**										
**< 60**	54	35	19	0.309	19	35	0.494	39	15	0.902
**≥ 60**	108	61	47		44	64		77	31	
**Gender**										
**Male**	115	65	50	0.267	47	68	0.419	81	34	0.605
**Female**	47	31	16		16	31		35	12	
**Tumor size**										
**< 5 cm**	88	51	37	0.712	39	49	0.122	59	29	0.160
**≥ 5cm**	74	45	29		24	50		57	17	
**Depth of tumor invasion**										
**T1-T2**	40	15	25	0.001	24	16	0.002	22	18	0.007
**T3-T4**	122	81	41		39	83		94	28	
**Histologic type**										
**Well and moderately differentiated**	91	47	44	0.026	42	49	0.032	61	30	0.144
**Poorly and undifferentiated**	71	49	22		21	50		55	16	
**TNM stage**										
**I**	19	4	15	0.001	14	5	0.004	7	12	0.001
**II**	20	10	10		10	10		14	6	
**III**	82	58	24		25	57		68	14	
**IV**	41	24	17		14	27		27	14	
**Lymph Nodes Metastasis**										
**No**	65	26	39	0.000	38	27	0.000	39	26	0.012
**Yes**	97	70	27		25	72		77	20	

**Table 3:** Correlations Associations between expression of Gal-1 and E-cadherin and vimentin expression in 162 human primary gastric cancer tissues

	Gal-1			
	Positive	Negative	*R*	*P-*value
**E-cadherin**				
**+**	6/162(3.70%)	57/162(35.19%)	−0.807	0.000
**-**	90/162(55.56%)	9/162(5.56%)		
**Vimentin**				
**+**	77/162(47.53%)	39/162(24.07%)	0.230	0.003
**-**	19/162(11.73%)	27/162(16.67%)		

**Figure 2:**
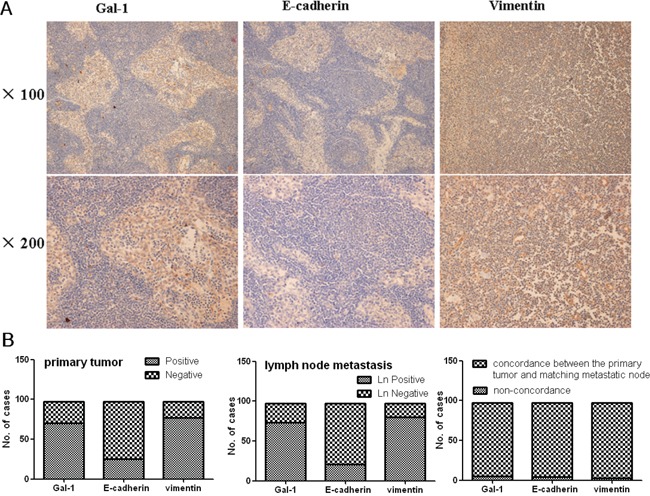
**A.** Immunohistochemical analysis of Gal-1, E-cadherin and vimentin expression in gastric cancer metastatic lymph node tissues. **B.** Gal-1, E-cadherin and vimentin expression in primary gastric cancer and the corresponding metastatic lymph node tissues, and the concordance in expression between the two sets of matched tissues. The strong correlation between primary tumors and matched lymph node metastases immunostaining expression was maintained even when primary tumors and matched lymph node metastases were categorized as positive/negative according to each individual staining marker cut-off level.

Nodal status is currently one of the most important prognostic factors in gastric cancer. We assessed the expression of Gal-1, E-cadherin and vimentin in metastatic lymph nodes from 97 patients with gastric cancer using immunostaining (Figure 2A). The expression of Gal-1 and these EMT markers in the lymph node metastases and matched primary tumors are summarized in Table 4 and Figure 2B. Compared to the primary tumors, the expression of E-cadherin reduced and that of vimentin increased in the matched metastatic lymph nodes. Bivariate Pearson correlation analysis demonstrated significant positive relationships between the expression of Gal-1 (*r* = 0.870, *P* < 0.000), E-cadherin (*r* = 0.892, *P* < 0.000) and vimentin (*r* = 0.905, *P* < 0.000) in the matched primary tumors and metastatic lymph nodes. When Gal-1 immunostaining was classified as positive/negative, only five (5.15%) of the 97 cases (Figure 2B), did not exhibit the same level of Gal-1 expression in the primary tumor and matching metastatic lymph node tissues; the respective levels of non-concordance for E-cadherin and vimentin were 4.12% (4/97) and 3.10% (3/97), respectively.

**Table 4:** Concordance between positive expression of Gal-1, E-Cadherin and vimentin in 97 human primary gastric cancer tissues and the corresponding metastatic lymph node tissues

Marker	Positive in Primary Tumor No. (%)	Positive in Lymph Nodes No. (%)	Non-concordance rate No. (%)
**Gal-1**	70/97 (72.16)	73/97 (75.26)	5/97 (5.15)
**E-cadherin**	25/97 (25.77)	21/97 (21.65)	4/97 (4.12)
**Vimentin**	77/97 (79.38)	80/97 (82.47)	3/97 (3.10)

### Gal-1 promotes the invasion of gastric cancer cells

**Figure 3:**
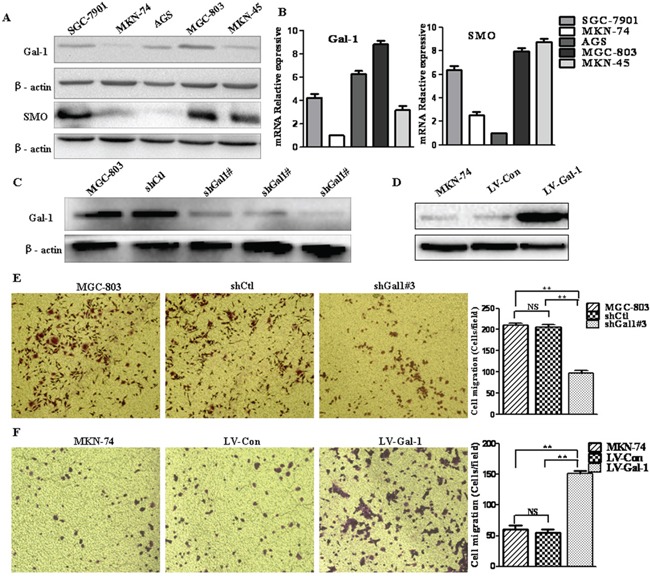
Gal-1 promotes the invasion of human gastric cancer cells **A** and **B.** Western blot and RT-PCR analysis of Gal-1 and SMO expression in SGC-7901, MKN-74, AGS, MGC-803 and MKN45 gastric cancer cells (*n* = 3). **C.** Western blot analysis of Gal-1 protein expression in control MGC-803 cells and cells infected with lentivirus carrying a control shRNA (shCtl) or different Gal-1 targeting sequences (shGal1#1, shGal1#2, shGal1#3) (*n* = 3). **D.** Western blot showing Gal-1 protein expression following overexpression of Gal-1 (LV-Gal-1) or the empty vector (LV-Con) in MKN-74 cells; β-actin served as a loading control (*n* = 3). **E.** Transwell invasion assay of control MGC-803, shCtl and shGal1#3 human gastric cancer cells. The number of invaded cells was quantified by counting in six randomly-selected fields at × 100 magnification; *n* = 3, **P < 0.01. **F.** Representative images of the invasive ability of MKN-74 cells infected with a lentiviral vector to overexpress Gal-1 (LV-Gal-1) or control lentiviral vector expressing GFP (LV-Con). Data are presented as mean numbers of invaded cells (magnification: ×100, *n* = 6, * *P* < 0.01, ** *P* < 0.01).

To confirm the relationship between Gal-1 and the metastasis of gastric cancer, we quantified Gal-1 expression in several gastric cancer cell lines, including AGS, MKN-45, SGC-7901, MKN-74 and MGC-803 cells (Figure 3A). Most human gastric cancer cell lines expressed high levels of Gal-1. Furthermore, MGC-803 cells, a high metastatic potential cell, showed the highest level of Gal-1 expression; whereas, the low metastatic potential cell line MKN-74 had the lowest expression of Gal-1 (Figure 3A). Moreover, the *Gal-1* mRNA levels detected in real-time PCR analyses were consistent with the protein levels determined by Western blotting (Figure 3B). Moreover, MGC-803 cells expressed higher levels of SMO than MKN-74 cells (Figure 3A and 3B). These results provided clear evidence that Gal-1 expression may correlate with the metastatic potential of gastric cancer cells.

Next, we assessed the ability of Gal-1 to promote the invasion of MGC-803 and MKN-74 gastric cancer cells using the in vitro Matrigel chamber-based invasion assay. As MGC-803 cells expressed the highest levels of Gal-1, we knocked down the expression of Gal-1 in this cell line. Cells were transfected with a shRNA control (shCtl) or three different Gal-1 shRNA sequences (shGal1#1, shGal1#2, shGal1#3); MGC-803 cells transfected with shGal1#3 (shGal1#3) efficiently reduced Gal-1 protein expression (> 90%; Figure 3C) and significantly reduced cell invasion ability compared to untransfected or shCtl-transfected MGC-803 cells (shCtl) (Figure 3E). Conversely, we stably expressed Gal-1 in MKN-74 cells using a lentiviral vector (LV-Gal-1) (Figure 3D). As shown in Figure 3F, overexpression of Gal-1 significantly increased the invasion capacity compared to control MKN-74 cells and MKN-74 cells transfected with the lentivirus control (LV-Con; Figure 3F). These observations indicate that Gal-1 may play a crucial role to promote gastric cancer cell invasion.

### Gal-1 regulates the transition between epithelial and mesenchymal phenotypes in gastric cancer cells

**Figure 4:**
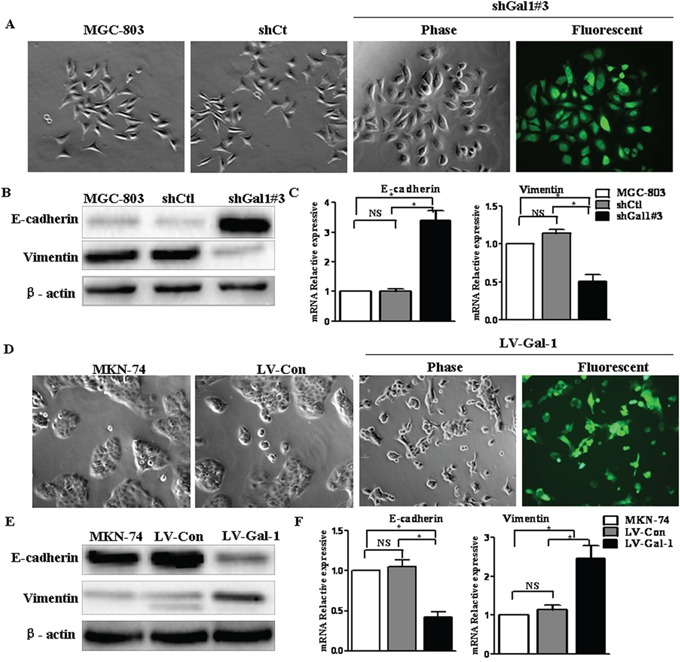
Gal-1 regulates the transition between epithelial and mesenchymal phenotypes in human gastric cancer cells **A.** Transfection of shGal1#3 induced morphologic changes in MGC-803 cells (magnification: ×100). **B.** E-cadherin and vimentin expression was analyzed in *Gal-1*-silenced gastric cancer cells by immunoblotting (*n* = 3). **C.** The mRNA expression levels of epithelial and mesenchymal markers were assessed in *Gal-1*-silenced MGC-803 cells by real-time RT-PCR analysis (*n* = 3, *P < 0.05). **D.** Overexpressing Gal-1 in MKN-74 (LV-Gal-1) cells resulted in a spindle-like morphology with larger gaps between cells, which were more elongated, compared to MKN-74 cells transfected with the lentivirus control (LV-Con) (magnification: ×100). **E** and **F.** Gal-1 overexpression markedly decreased E-cadherin and increased vimentin expression in MKN-74 cells (*n* = 3, *P < 0.05).

There is increasing evidence that metastasis is initiated by the EMT at the invasive front of primary carcinomas [21], and the EMT is recognized as critical step during tumor invasion and metastasis [22]. To examine whether Gal-1 could induce EMT-associated changes in gastric cancer cells, lentiviral-mediated delivery of shGal1#3 was used to knockdown Gal-1 expression in MGC-803 cells. Compared to cells infected with the control virus expressing luciferase shRNA (shCtl), knockdown of Gal-1 resulted in a more sheet-like architecture and less spindle-like fusiform shape (Figure 4A). The expression of the EMT markers E-cadherin and vimentin was quantified by real-time PCR and Western blot analyses at 48 h after transfection. Significantly increased mRNA and protein expression of the epithelial marker E-cadherin were observed in shGal1#3 cells, and conversely, the expression of the mesenchymal marker vimentin decreased significantly (Figure 4B and 4C). In contrast, LV-Gal-1 stably expressing Gal-1 exhibited a more spindle-like fusiform shape and less sheet-like architecture than control MKN-74 or LV-Con cells (Figure 4D). Simultaneously, both E-cadherin mRNA and protein expression reduced significantly and vimentin mRNA and protein levels significantly increased (Figure 4E and 4F). These results strongly indicate that Gal-1 functions as a driving force of the EMT in gastric cancer cells. Taken together, these findings suggest that Gal-1 plays an important role in regulating the EMT-MET plasticity of gastric cancer cells.

### Extracellular galectin-1 promotes invasion, the EMT and expression of Gli-1 in gastric cancer cells

MGC-803 cells and MKN-74 cells were treated with rGal-1 at concentrations of 1 μg/ml or 10 μg/ml; rGal-1 significantly increased the rate of invasion by MGC-803 and MKN-74 cells (Supplementary Figure S1A). Furthermore, the high concentration of rGal-1 significantly decreased the expression of E-cadherin and Gli-1 at both the mRNA and protein levels (*P* < 0.05; Supplementary Figure S1B). Simultaneously, vimentin mRNA and protein expression were significantly increased by rGal-1 (Supplementary Figure S1B and S1C). However, the expression of SMO and intrinsic Gal-1 did not significantly change in MGC-803 and MKN-74 cells treated with rGal-1 (Supplementary Figure S1B and S1C). Collectively, the above data indicates rGal-1 may promote gastric cancer progression through Gli-1, and that extracellular Gal-1 probably is an inducer of Gli-1 expression.

### Gal-1 regulates the EMT via activation of the Hh pathway in gastric cancer cells

**Figure 5:**
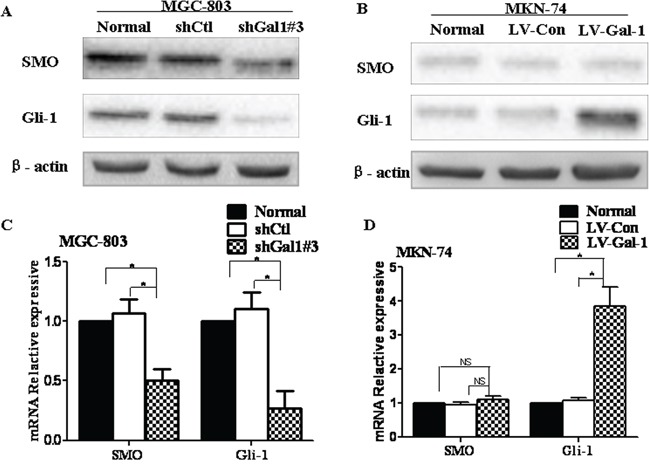
Gal-1 is associated with the Hedgehog pathway in human gastric cancer cells **A** and **B.** The protein expression levels of SMO and Gli-1 in *Gal-1*-silenced MGC-803 cells and Gal-1-overexpressing MKN-74 cells were evaluated by Western blotting (*n* = 3). **C** and **D.** The mRNA expression levels of *SMO* and *Gli-1* were quantified by RT-PCR in the same cells (*n* = 3, *P < 0.05).

It has been reported that the Hh pathway can lead to, or is required for, the EMT in gastric carcinomas [23]; therefore, we evaluated whether the Hh pathway is involved in the ability of Gal-1 to regulate the EMT in gastric cancer cells. Gal-1 was knocked down in MGC-803 cells by transfection of shGal1#3 or overexpressed in MKN-74/Gal-1 cells. The levels of Gli-1, an activator of target genes and itself a transcriptional target of the Hh pathway [24], were dramatically reduced in MGC-803 cells transfected with shGal1#3 (Figure 5A and 5C) and significantly increased in MKN-74/Gal-1 cells (Figure 5B and 5D). Interestingly, SMO expression levels decreased in cells transfected with shGal1#3, but were not affected in Gal-1-overexpressing cells. These data indicate that Gal-1 can activate the Hh pathway via Gli-1.

**Figure 6:**
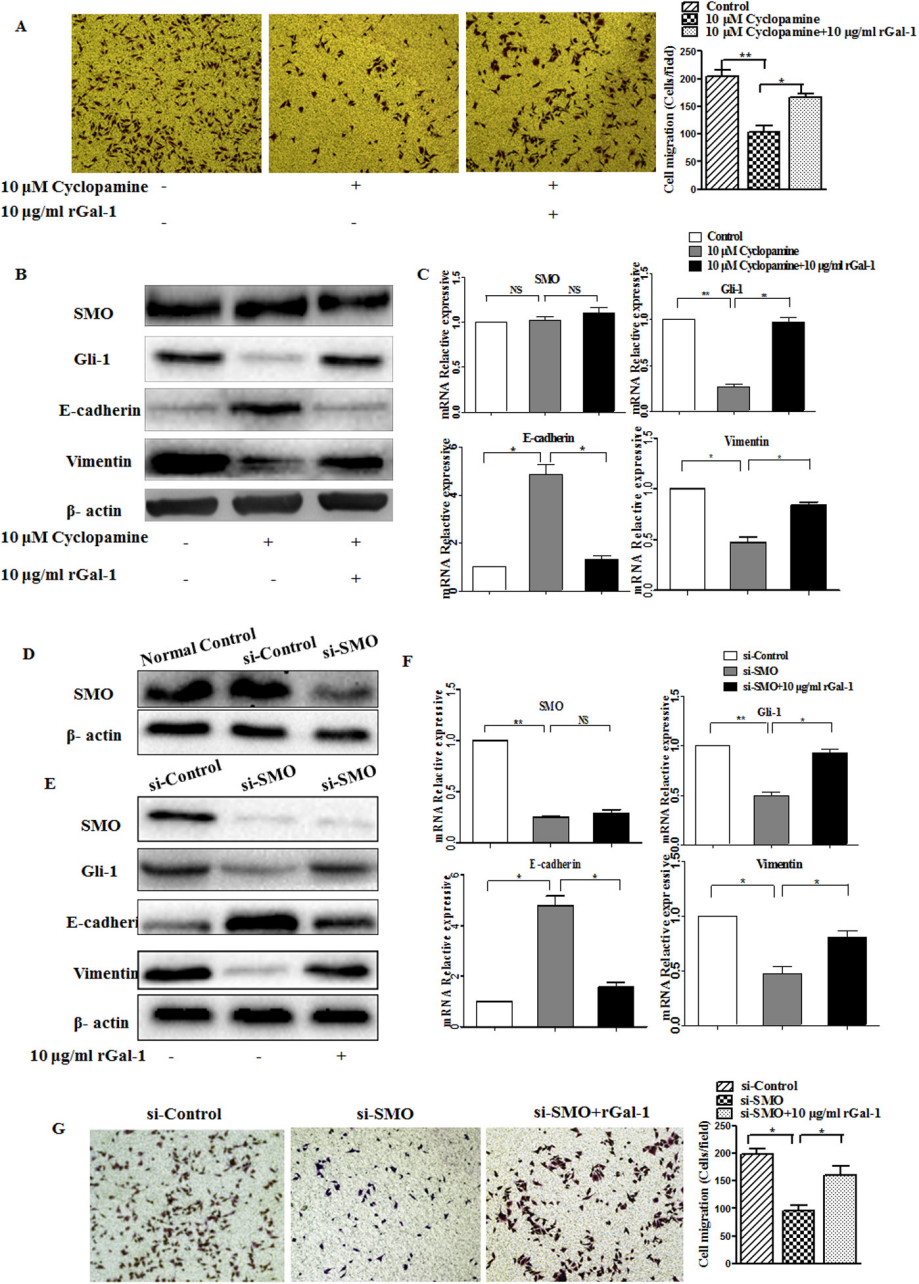
The effects of cyclopamine and the *SMO* siRNA on Gal-1-induced gastric cancer cell invasion and EMT **A.** Matrigel invasion assay of MGC-803 cells cultured with or without cyclopamine (10 μM) and recombinant Gal-1 (rGal-1; 10 μg/ml). The numbers of invaded cells were quantified in six randomly-selected fields at ×100 magnification (**P < 0.01, * *P* < 0.05). **B.** MGC-803 cells were cultured in the presence of the SMO inhibitor cyclopamine with or without rGal-1 for 48 h, then expression of the EMT-related molecules E-cadherin and vimentin and the Hh pathway-related proteins SMO and Gli-1 were analyzed by Western blotting. Untreated cells were used as a negative control (*n* = 3). **C.** Relative mRNA expression levels of E-cadherin and vimentin in cells cultured with the SMO inhibitor cyclopamine for 48 h in the presence or absence of rGal-1; * *P* < 0.05. **D.** After transfection, SMO protein expression was evaluated using Western blotting. The SMO siRNA completely inhibited SMO expression compared with the control siRNA transfected cells. **E, F** and **G.** MGC-803 cells before and after SMO knockdown seeded on plastic were treated with or without rGal-1 for 48 h, then SMO, Gli-1, E-cadherin and vimentin expression were analyzed by Western blotting or RT-PCR or the cells were subjected to the Transwell invasion assay (E, F). (G) Cell invasion was assessed using the Transwell cell invasion assay. Magnification: ×100, **P < 0.01, * *P* < 0.05.

To test whether Gal-1 activates the Hh pathway via a SMO-independent manner, we investigated the relationship between Gal-1-induced invasion and Hh pathway activation using the SMO antagonist cyclopamine and a siRNA targeting *SMO*. Neither cyclopamine at 10 μM nor the *SMO* siRNA affected the cell number at 24 h as indicated by the MTT assay. However, cyclopamine significantly decreased the invasive ability of MGC-803 cells (Figure 6A), reduced the expression of Gli-1 (Figure 6B and 6C), upregulated E-cadherin and downregulated vimentin (Figure 6B and 6C). However, these effects were significantly abolished in the presence of rGal-1. Furthermore, we used a siRNA to knockdown *SMO* in MGC-803 cells (Figure 6D). The *SMO* siRNA significantly reduced the expression of Gli-1 and vimentin (Figure 6E and 6F), upregulated E-cadherin (Figure 6E and 6F) and decreased the invasion of MGC-803 cells (Figure 6G). However, these effects were significantly abolished in the presence of rGal-1. Additionally, the presence of rGal-1 did not influence SMO expression (Figure 6E and 6F). Taken together, these data suggest Gal-1 increases gastric cancer cell invasion and promotes the EMT in a SMO-independent manner.

**Figure 7:**
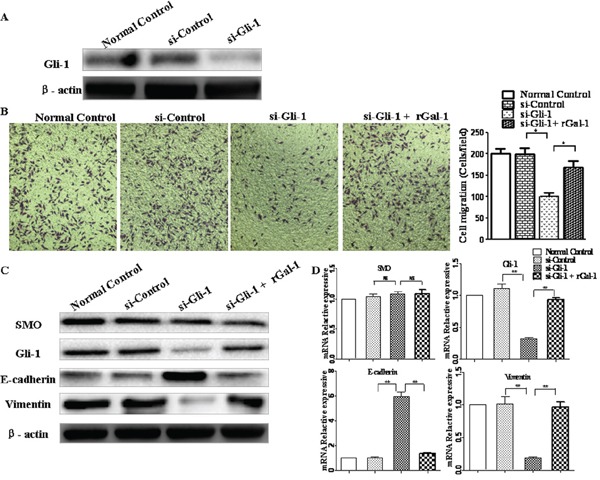
Gli-1 siRNA abolishes Gal-1-mediated invasion and EMT in gastric cancer cells **A.** Knockdown of *Gli-1* using siRNA was confirmed by Western blotting analysis at 48 h. **B.** Cell invasion in response to knockdown of *Gli-1*. At 48 h after transfection with siRNA, the cells were seeded into Matrigel-coated invasion chambers with or without rGal-1 for 24 h. Data are mean ± SD of three experiments. Magnification: ×100; **P < 0.01. **C** and **D.** Effects of *Gli-1* siRNA on the expression of SMO, E-cadherin and vimentin. At 48 h after transfection with siRNA, SMO, Gli-1, E-cadherin, and vimentin expression levels were determined by Western blotting and real-time RT-PCR; *P < 0.05.

To further confirm whether Gal-1 promotes Hh pathway activation via Gli-1, we used a siRNA to knockdown *Gli-1* in MGC-803 cells (Figure 7A). The *Gli-1* siRNA did not affect cell viability at 24 h, as indicated by the MTT assay. We found the invasion of MGC-803 cells significantly decreased (Figure 7B), both the mRNA and protein levels of E-cadherin obviously increased, and vimentin mRNA and protein expression decreased significantly, even in the presence of rGal-1 (Figure 7C and 7D). Additionally, in contrast to previous reports [25], knockdown of Gli-1 did not influence SMO expression. These results indicate that Gli-1 functions as an important downstream regulator of Gal-1 to regulate the EMT. Furthermore, Gal-1 induces the EMT in gastric cancer cells through non-canonical activation of the Hh pathway.

**Figure 8:**
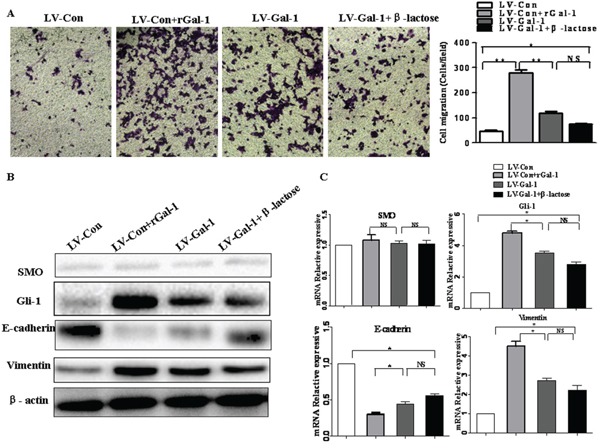
The effects of β-lactose on Gal-1-induced gastric cancer cell invasion and the EMT Effect of rGal-1, overexpressing Gal-1 using LV-Gal-1 and β-lactose on cell invasion ability **A.** and the expression of SMO, Gli-1, E-cadherin and vimentin **B** and **C.** in MKN-74 cells (Magnification: ×100; *P < 0.05, **P < 0.01).

To investigate the role of tumor-derived Gal-1, we compared rGal-1 treatment with LV-Gal-1-transfected cells. Compared to LV-Gal-1, treatment with rGal-1 increased the invasion capacity of MKN-74 cells (Figure 8A) and downregulated E-cadherin and upregulated Gli-1 and vimentin (Figure 8B and 8C). In addition, LV-Gal-1-transfected cells secreted only 0.86 ng Gal-1/10^6^ cells into the media. However, when MKN-74 cells were treated with 1 ng/ml rGal-1, significant changes in invasion and EMT marker expression were not detected (data not shown). In order to distinguish between the intracellular and extracellular activities of Gal-1 in LV-Gal-1 cells, we used β-lactose, a competitive inhibitor of Gal-1, to completely block the activity of exogenous Gal-1. Endogenous Gal-1 increased the invasion capacity of gastric cancer cells (Figure 8A). Treatment with 10 mM β-lactose did not significantly increase SMO, Gli-1, E-cadherin or vimentin expression (Figure 8B and 8C). Collectively, this study demonstrates that LV-Gal-1-transfected cells secrete a small amount of Gal-1 protein, and intracellular Gal-1 promotes the EMT by upregulating expression of Gli-1. These data indicate that overexpression of endogenous Gal-1 increases cell invasion and promotes the EMT via a SMO-independent manner.

## DISCUSSION

The development of gastric cancer is a complex, multi-step process associated with enormous numbers of genetic alterations, upregulation of cancer-causing genes, downregulation of tumor-suppressor genes, and acquisition of metastatic ability [26]. Hence, elucidation of the molecular mechanisms involved in gastric cancer has been the subject of extensive research in the last decade [27]. A high proportion of deaths related to gastric cancer are caused by tumor metastasis, postoperative recurrence, and delayed detection of advanced stage disease [28, 29]. However, effective diagnostic markers, drug targets and therapeutic strategies are still lacking, which prevents the successful treatment of gastric cancer. Our previous work demonstrated Gal-1 promotes gastric tumorigenesis and angiogenesis [30, 31]. The objective of this work was to further clarify the role of Gal-1 in gastric cancer.

This study confirms that, compared to the matched non-cancerous tissues, human gastric cancer tissues overexpress Gal-1. Moreover, overexpression of Gal-1 was significantly associated with the depth of tumor invasion, lymph node metastasis and advanced TNM stage. In addition, we observed strong positive correlations between the expression of Gal-1, E-cadherin and vimentin in the primary tumors and corresponding metastatic lymph nodes. Compared to the matched non-cancerous tissues, the expression of E-cadherin was downregulated in primary tumors, and decreased further in the lymph node metastases. Overexpression of Gal-1 in gastric cancer cells induced the EMT and promoted invasion in vitro. In contrast, silencing Gal-1 reversed these events in a gastric cancer cell line with high metastatic potential. In addition, we demonstrated a link between Gal-1 and the Hh pathway marker Gli-1; knockdown of Gli-1 attenuated the effects of Gal-1 and had similar effects to direct silencing of Gal-1. On the basis of these results, we propose a model by which Gal-1 promotes invasion and the EMT in gastric cancer via regulating Gli-1.

Gal-1, encoded by *LGALS1*, is a secreted protein that is overexpressed in both the stroma surrounding tumor cells and cancer-associated endothelial cells [8]. Although Gal-1 is normally present at the cell surface, it can also localize to the cell nucleus and cytoplasm and be secreted to the extracellular matrix [8]. Likewise, our previous work demonstrated Gal-1 was highly expressed in α-smooth muscle actin-positive cancer-associated fibroblasts in gastric cancer [30]. Gal-1 is linked to a variety of physiological cell functions; it has been shown to be important for tumor development and metastasis and has been associated with cell adhesion, invasion, angiogenesis and the immune response [8]. In addition, homodimeric Gal-1 promotes the adhesion of cancer cells to the extracellular matrix (ECM) and endothelial cells via carbohydrate-recognition domains [32]. It is intriguing that overexpression of Gal-1 was associated with increased invasiveness in oral squamous cell carcinoma [33] and cervical cancer cells [34] with low invasive potential, while siRNA-mediated inhibition of Gal-1 reduced the invasive ability of cancer cells with high invasive potential. Our work points to a novel function for Gal-1 in gastric cancer invasion by promoting the EMT via upregulation of Gli-1. Ectopic overexpression of Gal-1 in gastric cancer cells induced an EMT phenotype and stimulated invasion *in vitro*. Furthermore, these preliminary results not only indicate that Gal-1 promotes the EMT, but silencing Gal-1 leads to the MET. Taken together, these findings provide a mechanistic framework to explain our clinical observations that patients with gastric cancer whose tissue samples express high levels of Gal-1 have higher risks of distant metastasis and local relapse and significantly poorer overall survival [16, 31].

The cancer-associated EMT is a complex process that involves several related signaling pathways [5]. The Hh pathway is thought to be required for the EMT in carcinoma cells, including gastric cancer [23, 35]. Furthermore, abnormal activation of the Hh pathway and the presence of cells that have undergone the EMT can negatively influence the prognosis of patients with gastric cancer [36]. In an effort to shed light on the mechanism by which Gal-1 promotes the EMT in gastric cancer, we demonstrated the Hh pathway functions as an active signaling pathway during the Gal-1-induced EMT. To identify whether SMO or Gli-1 are directly regulated by Gal-1, we treated Gal-1-overexpressing gastric cancer cell lines with cyclopamine (a SMO antagonist) or a siRNA specific to SMO and *Gli-1* in the presence of rGal-1. Although blocking the function of SMO using cyclopamine or the siRNA suppressed expression of the transcription factor Gli-1 and dramatically inhibited cell invasion and the EMT, these effects were significantly abrogated in the presence of exogenous rGal-1. In contrast, exogenous rGal-1 could interrupt the ability of the *Gli-1* siRNA to suppress cell invasion and the EMT in gastric cancer cells. In addition, exogenous rGal-1 was a more powerful inducer of Gli-1 expression, and this effect could not be abrogated by the *Gli-1* siRNA. Furthermore, expression of SMO was also not affected by addition of exogenous rGal-1. To investigate the role of intracellular Gal-1 in these processes, we used β-lactose to successfully inhibit the binding of extracellular Gal-1 to its receptor. Therefore, intracellular Gal-1 is necessary for the EMT and invasion via non-canonical Hh signaling.

**Figure 9:**
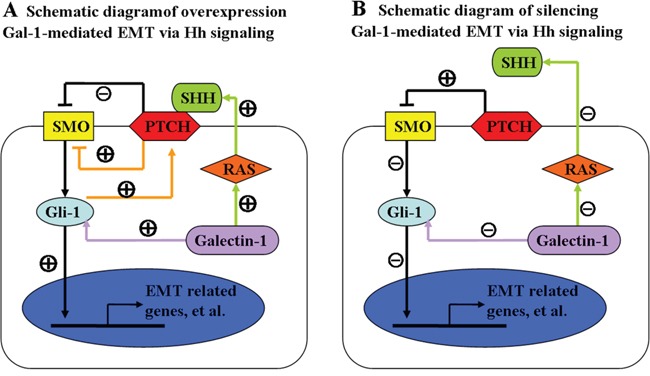
Schematic diagram of the relationships between Gal-1, the Hedgehog pathway and the EMT Gal-1 (purple arrow) can modulate the non-canonical Hedgehog pathway by increasing the transcription of *Gli-1* without the altering the expression of sonic hedgehog (SHH) or smoothened (SMO). Accumulated Gli-1 translocates to the nucleus, resulting in activation of EMT-related genes, leading to the EMT switch in cancer cells. **A.** Earlier studies suggested autocrine signaling by the SHH ligand was functionally important in the context of Ras activation (green arrow) in gastric cancer cells. SHH signaling in cancer cells was thought to occur via canonical hedgehog signaling, whereby SHH binds to Ptch and induces SMO signaling resulting in activation of Gli-1. Previous studies demonstrated Gli-1 can be upregulated by Ptch in the presence of SHH, which establishes a negative feedback loop (orange arrow). Silencing Gal-1 **B.** abrogated these effects, including the negative feedback loop and the ability of SHH to bind to PTCH resulting in activation of SMO.

Hh signaling is commonly classified as either canonical or non-canonical, and SMO-independent activation of Gli-1 is termed non-canonical Hh signaling [18]. Accordingly, we concluded that SMO-independent activation of Gli-1, i.e., non-canonical Hh signaling, was at least partially responsible for Gal-1-induced EMT and invasion in gastric cancer cells. These data suggest Gal-1 induces the EMT in gastric cancer by upregulating Gli-1. However, a recent study reported that after Hh signaling activation, Gli-1 can upregulate Ptch the transmembrane receptor of SHH. PTCH inhibits SMO, which activates Gli-1, in turn establishing a negative feedback loop in the presence of SHH signaling [37–39]. Another study found that RAS signaling can induce or enhance SHH expression [40]. Moreover, Kloog et al. reported that intracellular Gal-1 is a major regulator of H-Ras nanoclusters, which contributes to Ras membrane anchorage and cell transformation [41, 42]. Recently, Blazevits et al. suggested Gal-1 also engages K-ras effectors besides H-Ras nanoclusters [43]. In agreement with these published observations, we demonstrated that downregulating Gal-1 reduced SMO and Gli-1 expression, whereas overexpressing Gal-1 increased only Gli-1 expression. These observations suggest Gal-1 activates the RAS pathway and leads to increased SHH protein expression to positively regulate the expression of SMO, while a negative feedback loop in the presence of SHH signaling exerts a negative effect on SMO expression. The two effects cancel each other out, resulting in constant expression of SMO (Figure 9A). Intriguingly, loss of Gal-1 in gastric cancer cells resulted in a lack of SHH expression, leading to Hh inactivation, which downregulated SMO and Gli-1 (Figure 9B). Therefore, for the first time, this study demonstrates the existence of crosstalk between Gal-1 and the Hh pathway during gastric cancer invasion and the EMT. However, more investigation is are required to explore the internal links between Gal-1 and Gli-1.

In conclusion, this research demonstrates Gal-1 plays an important role in human gastric cancer invasion and metastasis. Specifically, we provide the first evidence to indicate Gal-1 functions in an autocrine manner to induce the EMT in gastric cancer cells *in vitro*, and also enhances the tumorigenic and metastatic capacities of gastric cancer cells *in vivo*. Mechanistically, our results suggest that cross-talk between Gal-1 and the Hh/Gli pathway could play an important role in gastric cancer invasion and the EMT (Figure 9). These findings not only improve our understanding of the molecular mechanisms underlying the effects of Gal-1 in gastric cancer metastasis, but also provide new insight into Gal-1 as an important therapeutic target associated with gastric cancer metastasis.

## MATERIALS AND METHODS

### Patient selection and tissue preparation

From January 2012 to August 2012, 162 patients with gastric cancer were treated at the Department of Gastrointestinal Surgery, Clinical Medical College of Yangzhou University (Subei People's Hospital of Jiangsu Province). The clinicopathological features of these patients are shown in Table 2. All patients underwent radical resection; no patients received either chemotherapy or radiotherapy before surgery. This study followed the tenets of the Declaration of Helsinki, and informed written consent was obtained from all patients and controls after clinicians explained the purpose, nature and possible consequences of the study. The study protocol was approved by the Medical Ethics Committee of First Clinic Medical School of Yangzhou University (YZU-EC-JS2352).

### Reagents and antibodies

The pharmacological reagent cyclopamine was provided by Selleck Chemicals (Houston, TX, USA). Recombinant human Gal-1 (rGal-1) was obtained from Peprotech (Rocky Hill, NJ, USA), and dissolved in 0.1% bovine serum albumin (BSA; Biosharp, Anhui, China). Anti-galectin-1 antibody (Santa Cruz Biotechnology, Santa Cruz, CA, USA), anti-SMO antibody (Abcam, Cambridge, UK), anti-Gli-1 antibody (Abcam), anti-SHH antibody (Abcam), anti-E-Cadherin antibody (Cell Signaling Technology, Danvers, MA, USA), anti-Vimentin (Cell Signaling Technology), anti-β-actin antibody (Beyotime, Jiangsu, China), HRP-conjugated goat anti-mouse IgG and HRP-conjugated goat anti-rabbit IgG (Santa Cruz Biotechnology) were used in this study.

### Immunohistochemistry

Immunohistochemical staining of human paraffin-embedded gastric cancer and normal tissue sections was carried out as previously described with minor modifications [16]. Briefly, after antigen retrieval, slides were incubated with primary antibodies against E-cadherin, vimentin, galectin-1 or Gli-1 overnight at 4°C, followed by incubation with biotin-conjugated secondary antibodies, then horseradish peroxidase-conjugated streptavidin. The sections were stained with DAB and counterstained with hematoxylin. Negative controls were treated identically, though the primary antibodies were omitted. Staining density was scored using standard methods, as described previously [16]: negative staining was defined as negative (no visible staining) or weak staining (light brown staining in < 20% of tumor cells); positive staining as moderate or strong staining (brown or dark brown staining in > 20% of tumor cells).

### Cell lines and culture conditions

The human gastric cancer lines AGS (moderately-differentiated), MKN-45 (poorly-differentiated), SGC-7901 (moderately-differentiated), MKN-74 (well-differentiated) and MGC-803 (poorly-differentiated) were purchased from the Type Culture Collection of the Chinese Academy of Sciences (Shanghai, China). MGC-803 and AGS cells were maintained in Dulbecco's modified Eagle's medium (DMEM; Hyclone, Logan, Utah, USA) supplemented with 10% FBS and the other cell lines were cultured in Roswell Park Memorial Institute (RPMI) 1640 medium (Hyclone) containing 10% FBS. All cells were maintained at 37°C in a humidified atmosphere containing 5% CO2. Before experiments, gastric cancer cells in log phase growth in six-well plates were cultured in media containing only 1% FBS for 24 h.

### Lentiviral production and transduction

Human *LGALS1* (GenBank accession numberNM_002305) was inserted into the GV248 and GV358 lentiviral vectors (Genechem, Shanghai, China) to silence and upregulate the expression of Gal-1, respectively. The three shRNA sequences were as follows: Gal-1 sh1 (5'-CCGGCACCATCGTGTGCAACAGCAACTCGAGTTGCTGTTGCACACGATGGTGTTTTTG-3′); Gal-1 sh2 (5'-CCGGCCAGCCTGGAAGTGTTGCAGACTCGAGTCTGCAACACTTCCAGGCTGGTTTTTG-3′); Gal-1 sh3 (5'-CCGGGCTGCCAGATGGATACGAATTCTCGAGAATTCGTATCCATCTGGCAGCTTTTTG-3′). GV248 and GV358 lentiviral vectors were constructed to silence and upregulate the expression of LGALS1; a negative control vector containing the cytomegalovirus (CMV) promoter and expressing high levels of green fluorescent protein (GFP) was also created. The negative control was also created from GV248 and GV358. The lentiviral vectors were transfected into MGC-803 and MKN-74 cells at a multiplicity of infection (MOI) ranging from 1 to 100 in the presence of 5 μg/ml polybrene (Sigma-Aldrich, St. Louis, MO, USA). To produce stably transfected cell lines, the cells were cultured in the presence of puromycin (Sigma-Aldrich). The cells were used for subsequent experiments after the expression of the target gene was confirmed using Western blotting.

### Western blot analyses

Total lysates of treated cells were prepared using RIPA buffer containing 1× Tris-buffered saline, 1% Nonidet P-40, 0.5% sodium deoxycholate and 0.1% sodium dodecyl sulfate. Total proteins (50 μg) from each lysate were separated by SDS/PAGE and transferred onto PVDF membranes, and then probed with the indicated antibodies using standard protocols.

### RNA extraction and real-time PCR

Real-time PCR was performed to determine the mRNA expression levels of *LGALS1, SMO, Gli-1*, E-cadherin and vimentin. Total RNA was extracted using TRIzol reagent (Invitrogen, Carlsbad, CA, USA) following the manufacturer's instructions. First-strand reverse transcription was performed using the PrimeScript RT reagent Kit (TaKaRa, Dalian, China). The real-time PCR analyses were conducted on an iQ5 Multicolor Real-Time PCR Detection System (Bio-Rad, Hercules, CA, USA) using SYBR Green Real-time PCR Master Mix (TaKaRa). The PCR program was 30 s at 95°C followed by 40 cycles at 95°C for 5 s, 60°C for 30 s and 72°C for 30 s. Glyceraldehyde phosphate dehydrogenase (*GAPDH*) was used as the reference control. Fold changes in the mRNA levels of target genes were calculated relative to *GAPDH*. All results are reported as the average ratios of three different independent experiments. The following primers were used: *Gal-1* (forward): CTGGAAGTGTTGCAGAGGTGT and (reverse) CTGGCTGATTTCAGTCAAAGG; SMO (forward) CAGGTGGATGGGGACTCTGTGAGT and (reverse) GAGTCATGACTCCTCGGATGAGG); *Gli-1* (forward) GGGATGATCCCACATCCTCAGTC and (reverse) CTGGAGCAGCCCCCCCAGT; E-cadherin (forward) TCGTCACCACAAATCCAGTG and (reverse): CATTCACATCAAGCACATCC); vimentin (forward) TGAATACCAAGACCTGCTCAA) and (reverse) ATCAACCAGAGGGAGTGA ATC); and GAPDH (forward) TGACTTCAACAGCGACACCCA) and (reverse) CACCCTGTTGCTGTAGCCAAA.

### Transfection of siRNA

The siRNA against *Gli-1* and a negative control siRNA were purchased from Genechem (Shanghai, China). MKN-74 cells seeded in six-well plates were transfected with control or *Gli-1* siRNA using Lipofectamine 2000 (Invitrogen, Carlsbad, CA, USA) according to the manufacturer's instructions. The cells were harvested for further experiments at 24 h after transfection.

### Matrigel invasion assay

Gastric cancer cell invasion was assessed using a chamber-based invasion assay. In brief, the upper surfaces of the 24-well Transwell inserts (pore size, 8.0 μm; Corning, New York, USA) were coated with 150 mg Matrigel basement membrane (BD Biosciences, San Diego, CA, USA). The cells were re-suspended in serum-free RPMI or DMEM medium, then cell suspensions (100 μl containing 10,000 cells) were seeded onto the filters in 24-well chambers; 500 μl of medium containing 10% FBS was placed in the lower chambers as a chemoattractant. The cells were allowed to migrate for 24 h at 37°C. Cells remaining on the upper surface of the membrane were removed using a cotton swab. The filters were fixed with 4% paraformaldehyde, and the cells were stained with 0.05% crystal violet solution. The cells that had migrated from the upper to the lower side of the filter were counted under a light microscope in 10 randomly-selected fields at 100× magnification. Tumor cell invasion assays were performed in triplicate.

### Statistical analyses

Protein expression levels and clinicopathological features were compared using the λ^2^-test. Other data is presented as the mean ± standard error values. One-way ANOVA with the Least Significant Difference (LSD) post hoc test was used for multiple comparisons using SPSS version 13.0 software (SPSS, Chicago, USA). *P*-values < 0.05 were considered significant.

## SUPPLEMENTARY FIGURE


