# Supplementary material for: Galectin-1 induces invasion and the epithelial-mesenchymal transition in human gastric cancer cells via non-canonical activation of the hedgehog signaling pathway
Source: Oncotarget. 2016 Nov 8;7(50):83611–26. doi: 10.18632/oncotarget.13201 (PMC5347792; doi:10.18632/oncotarget.13201)
Supplement: Supplementary file 1 [file oncotarget-07-83611-s001.pdf]

Galectin-1 induces invasion and the epithelial-mesenchymal transition in human gastric cancer cells via non-canonical activation of the hedgehog signaling pathway

SUPPLEMENTARY FIGURE

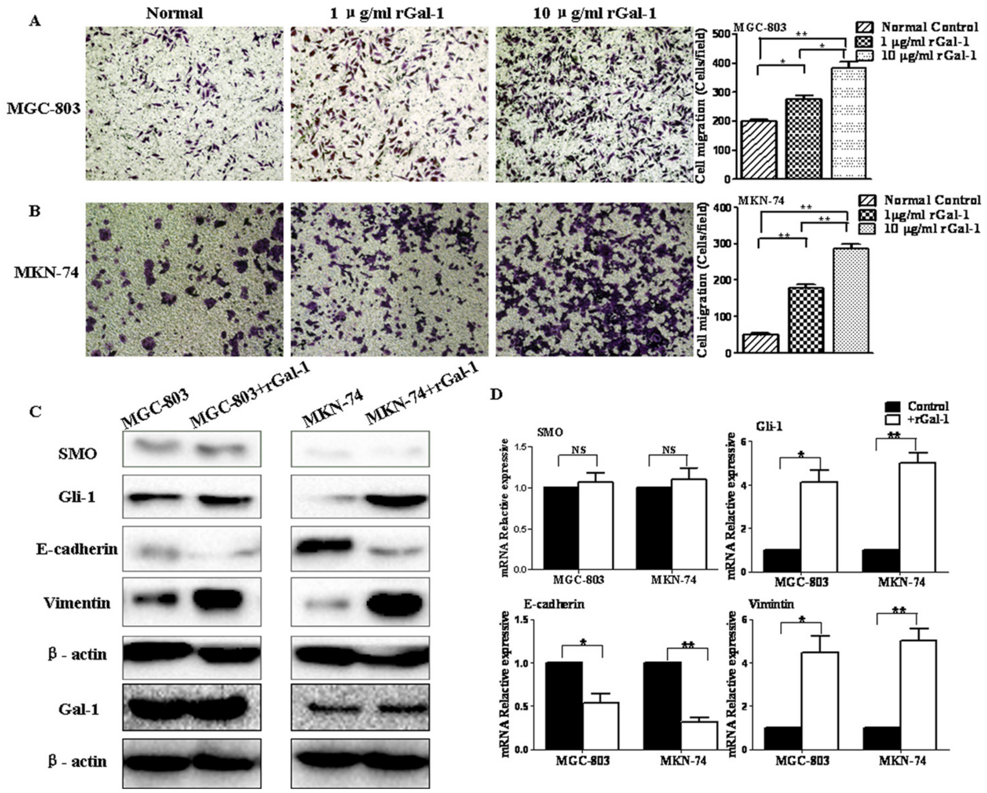

Supplementary Figure S1: Modulation of recombinant Gal-1 influences the invasive ability of gastric cancer cells. **A** and **B.** Effects of rGal-1 on gastric cancer cell invasion in matrigel invasion assay. The number of invaded cells was quantified by counting the number of cells from three randomly-selected fields. **C** and **D.** GC-803 and MKN-74 cells were treated with 10 μg /ml rGal-1 for 48 h prior to harvest; normal media was used as a negative control. Whole cell protein and RNA extracts were subject to Western blotting (C) or real-time RT-PCR (D) analysis of SMO, Gli-1, E-cadherin, vimentin and Gal-1 expression; β-actin was used as an internal loading control. Magnification: ×100; $*P < 0.05$, $**P < 0.01$.
